# SGLT2 Inhibitors In Older Adults With Cardiovascular Disease: A Systematic Review And Meta‐Analysis

**DOI:** 10.1093/geroni/igaf122.2552

**Published:** 2025-12-31

**Authors:** Kota Minami, Rika Terashima, Yuji Yamada, Satoshi Miyashita

**Affiliations:** Saga University, Saga, Saga, Japan; Stanford University, Stanford, California, United States; Icahn School of Medicine at Mount Sinai, New York, New York, United States; Cleveland Clinic Foundation, Cleveland, Ohio, United States

## Abstract

SGLT2 inhibitors have shown significant cardiorenal benefits, leading to their use in various cardiovascular conditions. However, there is limited data on their effectiveness in older adults. This systematic review and meta‐analysis of retrospective cohort studies (RCTs) published between January 2015 and January 2025 used MEDLINE. Eligible studies reported hazard ratios (HR) for cardiovascular outcomes in adults aged ≥65 years with cardiovascular disease. The primary outcome was a composite of hospitalization for heart failure (HHF), urgent HF visits, and cardiovascular death (CVD), while secondary outcomes included all-cause mortality, CVD, and HHF individually. Subgroup analyses were conducted for the primary endpoint in studies enrolling patients with HF, T2DM, adults aged 65-74 years, and those ≥75 years, and by SGLT2 inhibitor agent type. Nine RCTs were included. SGLT2 inhibitors significantly reduced the composite outcome (HR 0.75, 95% CI 0.67–0.83, I² = 51%), all-cause mortality (HR 0.80, 95% CI 0.66–0.97, I² = 68%), CVD (HR 0.78, 95% CI 0.65–0.94, I² = 61%), and HHF (HR 0.73, 95%CI 0.65–0.83, I² = 0.0%). Subgroup analyses showed consistent benefits in patients with HF (HR 0.76, 95% CI 0.70-0.82, I² = 10%), T2DM (HR 0.65, 95% CI 0.44-0.95, I² = 81%), and those ≥75 years (HR 0.71, 95% CI 0.57-0.88, I² = 51%). No significant difference was found between SGLT2 inhibitor subgroups (p = 0.09). These results support the use of SGLT2 inhibitors in older adults with cardiovascular disease, with careful monitoring for age-related risks.

